# Adolescent’s movement behaviors and built environment: a latent class analysis

**DOI:** 10.1186/s12889-021-11974-4

**Published:** 2021-10-25

**Authors:** Isabella Toledo Caetano, Valter Paulo Neves Miranda, Fernanda Karina dos Santos, Paulo Roberto dos Santos Amorim

**Affiliations:** Post-graduate Program in Physical Education, Federal University of Viçosa, Peter Henry Rolfs Avenue, s/n, University Campus, Viçosa, Minas Gerais Brazil

**Keywords:** Built environment, Neighborhood, Accelerometer, Movement behaviors, Latent class analysis

## Abstract

**Background:**

Latent class analysis (LCA) is an alternative and innovative approach to verify the relation of the various combinations of the constructed environment and movement behavior (levels of physical activity, sedentary behavior, and sleep) characteristics. This study aimed to identify latent classes based on the characteristics of the neighborhood environment perceived by adolescents and their association with gender, socioeconomic status (SS), body composition and movement behaviors.

**Methods:**

This cross-sectional study includes 309 Brazilian adolescents (14 to 16 years old, 57% female). The characteristics of the neighborhood environment perceived were analyzed by the Neighborhood Walkability for Youth Scale*.* Accelerometers were used for a week to evaluate the movement behaviors. Questionnaires assessed the screen times, total sitting time (TST), and sociodemographic characteristics. LCA was used for modeling the “Perceived Enviroment” variable, having been conducted in the poLCA (Polychromous Variable Latent Class Analysis) package of the R statistical software.

**Results:**

Three classes were recognized: class 1, “Best Perceived Environment” with 23.03% of adolescents; class 2, “Moderate Perceived Environment”, 63.33%; and class 3, “Worst Perceived Environment”, 13.67%. Light physical activity (LPA), TST, and SS were associated with class prevalence. The adolescents with medium and low SS were, respectively, 3.42 (95% CI 1.62–7.21) and 4.18 (95% CI 1.66–10.50) more likely to belong to class 2, and those with low SS were 5.21 (95% CI 1.35–20.13) more likely to belong to class 3. Class 1 adolescents were associated with a lower chance (OR: 0.09, 95% CI 0.02–0.55) of involvement in ‘adequate LPA time’ compared to class 3. Class 1 adolescents were associated with a lower chance (OR: 0.31, 95% CI 0.12–0.79) of involvement in ‘adequate TST’ compared to class 2. There was a difference between the LPA and TST classes; class 3 presented a longer time in LPA than class 1; class 1 had higher TST than the other classes.

**Conclusion:**

The findings highlight the influence of neighborhood classes on adolescents’ LPA and TST.

**Supplementary Information:**

The online version contains supplementary material available at 10.1186/s12889-021-11974-4.

## Introduction

Current public health recommendations establish that adolescents should accumulate at least 60 min of moderate-to-vigorous physical activity (MVPA) per day [1, 2]. Despite these recommendations, studies show that most adolescents do not accumulate the minimum recommended MVPA time [2–4]. A recent study conducted by Guthold et al. [4] using a grouped analysis of 298 population-based studies from 146 countries, representing 1.6 million adolescents between 11 and 17 years old, showed that 81% of the sample does not reach the physical activity (PA) recommendations. In Brazil, the evaluation of more than 54,000 adolescents between the ages of 14 and 17 in the Cardiovascular Risks in Adolescents Study (Portuguese acronym, “ERICA”) showed that approximately 56% of adolescents do not reach 300 min of PA per week. In contrast, 30% do not perform any PA [3]. Additionally, an increase in the prevalence of sedentary behavior (SB) has been observed, mainly related to screen time (ST) in this population [5]. ST refers to the sum of time spent in front of any type of electronic media, including television, video games, smartphones and tablets [6]. Thus, recent investigations have focused on unveiling the roles of movement behaviors (PA, SB, and sleep) in adolescent health [7–9]. However, it is important to highlight that the results of these surveys that assess the relation between movement behaviors, ST and adolescent health outcomes have been based on independent approaches, and does not consider the probability that the individual is simultaneously involved in different behaviors [10], for example, watching television while running on the treadmill. Thus, it does not consider the integration of different behaviors in the final outcome [10], nor does it consider the intrinsic co-dependence existing between movement behaviors in the finite time of the day [11].

The determinants of participation in PA are multifaceted and include several factors that arise from intrapersonal characteristics (biological, psychological, demographic) [12–14], interpersonal (social, cultural) [15], environmental (built, natural, social) [16, 17], organizational and political [12, 13], which demonstrates the complexity and diversity of aspects that can influence this practice.

Theoretical models have reinforced the importance of physical environment characteristics for the adoption of physically active behavior [13, 18]. The premise is that since people practice PA in a physical space, specific characteristics of the built environment are fundamental to encourage the PA and pattern development [18–20]. Physical environments include natural environment features, such as geography and weather conditions, as well as the built environment’s characteristics, including the design of the neighborhoods and available resources such as structures, green spaces, buildings, and objects, which are created or altered by man [18, 21, 22]. All of these can have a specific influence in each PA context or domain (transportation, occupation, leisure, household chores) [23, 24].

Research on the association between environmental factors and adolescents PA has increased in recent years [25]. Some recent systematic reviews [16, 17, 26] that link aspects related to the built environment and children and adolescent’s participation in different areas of activities, including PA, recreational activities, and resources that facilitate walking, show that there is a wide variety of environmental attributes between studies. In these reviews [16, 17, 26], the most investigated environmental attributes have been: access to recreational facilities, residential density, traffic, combination of land use, presence of parks and playgrounds, crime, neighborhood aesthetics, distance perception until leisure facilities, the quality of green spaces, parks and sidewalks. In addition, some studies show that favorable physical environments, such as green space near the house [27], with greater walking ability in the neighborhood of the house [28], higher residential density, better diversified land use, with paved and connected streets, safer against crimes, closer to parks and commerce [28–31] are associated with longer PA and MVPA in adolescents. In Brazil, a recent study conducted with adolescents examined the association between objectively measured PA and several perceived characteristics of the built environment and observed that only four attributes, such as living in front of the beach, street lighting, paved streets, and bicycle paths, were associated with this population’s PA patterns [25], which demonstrates that different environmental characteristics can have distinctive effects in the PA.

Thus, the neighborhood environment in which adolescents live can attenuate or accentuate activity-related behaviors, as well as the association of this relation with other outcomes, such as obesity and socioeconomic status. For example, access to suitable places to play, positive patterns for PA in the neighborhood and the high perceived security that mothers have about the neighborhood have an inverse relation with ST [32], adolescents living in most favorable neighborhoods to practice PA, spent less time with SB [30]. On the other hand, neighborhoods with few commercial businesses, low socioeconomic status and less security had a significantly higher proportion of obese adolescents [33]. In addition, a different perception between the genders about the neighborhood environment may reflect on the PA levels. For example, Lopes et al. [34] observed a more positive perception of the neighborhood environment among boys than among girls, which was associated with greater PA practice among boys compared to girls. However, in Brazil, there are still little evidence regarding the relation between the neighborhood environment and activity-related behaviors, as well as the association of this relation with gender, body composition and socioeconomic status.

A characteristic of studies of this nature is that many evaluate the neighborhood’s individual characteristics and its relations with PA [22]. Still, few studies analyze the neighborhood’s characteristics as a whole [35]. It is common for some neighborhoods in urban areas to have a great combination of land use, street connectivity, and residential density. In contrast, in other parts of the same city, neighborhoods have different combinations of the same characteristics [36]. Thus, examining how neighborhoods work as a whole, classifying them based on environmental classes can be interesting. Besides, the combinations of environmental characteristics offer a more comprehensive approach on identifying the influence of PA’s built environment [29, 37].

An alternative and innovative method to classify the neighborhood according to a set of environmental characteristics is latent class analysis (LCA), which is related to a specific type of cluster analysis, called multivariate mixture estimation [38, 39]. Based on this method, the latent variable created will jointly represent the surveyed population’s neighborhood environment characteristics [20, 37]. LCA has been used in studies to assess the effect of the built environment measured objectively (georeferenced data – GIS) and/or subjectively (perceived measures – questionnaires) on PA. For example, McDonald et al. [20] used the LCA to classify the neighborhood of 344 adolescents based on georeferenced data from the environment, then verified whether the PA and SB differed between classes. The LCA was also used in the International Prevalence Study (IPS) to create neighborhood classes from 20 countries from subjective information and link them to adult PA [37]. Additionally, it was possible to observe that all identified studies that involved LCA and environment were conducted in large urban centers and generally investigate total PA or MVPA and total SB.

However, no study explored the associations of neighborhood classes through LCA using perceived environmental characteristics with different PA levels and different expressions of SB in adolescents as far as we could verify. It is also essential to understand how these configurations of combinations of characteristics from the neighborhood environment can interfere with adolescents’ movement behaviors in smaller cities. Thus, this study aimed to identify latent classes based on the characteristics of the environment constructed by the neighborhood perceived as adolescents in a countryside city, as well as its association with gender, socioeconomic *status,* body mass index, and movement behaviors.

## Methods

### Study design and participants

This cross-sectional study was conducted between March and December 2019, with a random and representative sample of adolescents of both sexes, ages 14 to 16, regularly enrolled in the first year of high school in public schools (five state schools and one federal) of the city Viçosa, Minas Gerais, Brazil. Viçosa is considered a small countryside town [40], with an estimated population of 78.846 inhabitants in 2019 [41].

Following the Declaration of Helsinki guidelines, the study protocol was conducted and approved by the Research Ethics Committee involving human beings of the Federal University of Viçosa, under approval number 00925118.6.0000.5153. Before taking any action, the participants and their parents or legal guardians signed the Informed Consent Forms.

A specific formula from the EpiInfo software, version 7.2.2.16, was used to determine the sample size for cross-sectional studies (Georgia, USA). The population size was set at 968 (total number of students enrolled in the first year of high school in the city’s public schools) and the prevalence of results at 50% since the study considers different movement behaviors of the adolescent population [42, 43]. The 50% prevalence value was previously adopted in a study with high school students from Curitiba (PR), Brazil [34] to verify the association between perceived neighborhood environment and physical activity (PA). The acceptable level of error was set at 5%, the confidence level at 95%, and a drawing effect of 1.1. With these configurations, a minimum sample size of 305 adolescents was found. 20% were added to this calculation to recover possible losses, totaling 367 adolescents. Then, 6 schools were selected to participate. Students from each school were selected by drawing lots, based on the list of enrolled students [44]. Thus, everyone had the same chance of participating in the sample.

To be included in the study, adolescents should be between 14 and 16 years of age, return signed consent and agreement form, and be regularly enrolled in the first year of high school. Exclusion criteria included pregnancy, participating in a program for weight reduction or control, temporary or permanent physical or mental disability, and regular use of diuretics/laxatives.

The evaluations were carried out by a team of evaluators previously trained. The data collection occurred in four meetings with each participant. In the first meeting, the adolescents received information about the research and its procedures, were invited to participate and received the consent forms. In the second meeting, which lasted an average of 60 min, the adolescents delivered the signed forms and filled out the research questionnaires, in the classroom, with the help of the first author of the study. Before filling out the questionnaire, the participants received a prior explanation of the questionnaires, were instructed to sit away from each other to maintain privacy while answering and were asked to answer honestly. In the third meeting, held in private rooms provided by the schools, that lasted approximately 30 min, anthropometric evaluations and accelerometer placement (which should be used for eight consecutive days) were performed for the direct PA and SB assessment. A verbal explanation of the use of the device was provided along with an instructions sheet and an equipment usage journal that should be filled in at the moments when the monitor was removed from the body and night sleep times. In this same meeting, a socioeconomic questionnaire that should be filled out by parents or legal guardians was dispensed to each student to take home. Students should return with the completed questionnaire and deliver the accelerometer along with the usage journal in the fourth meeting.

### Neighborhood environment

The neighborhood environment was evaluated based on the *Neighborhood Walkability Scale for Youth (*NEWS-*Y)* [45], adapted for Brazilian adolescents [46]. NEWS-Y was developed to evaluate the perception of the neighborhood environment’s characteristics that may be associated with walking and other types of PA [47, 48]. The scale’s questions are presented in 8 domains regarding the adolescents’ perception of land use mix-diversity, street connectivity, land use mix-access, walking/cycling facilities, pedestrian and automobile traffic safety, residential density, neighborhood aesthetics and crime safety [45].

All questions are related to the adolescents’ residence surroundings characteristics, considering the traveled distance of 10 to 15 min on foot. Residential density was obtained through the perception of the predominant types of residences in the neighborhood, by a *Likert* scale of 5 points, ranging from “none” to “all”. Items related to the land use mix-diversity measured the walk time from home to 34 facilities/establishments (e. g. school, clubs, shops, parks), on a 5-point scale, ranging from ‘1–5 min’ to ‘31 min or more’. A *Likert* scale of 4 points was used for all other questions, with options ranging from “totally disagree” to “totally agree”. An average was calculated for each of the domains, so that values greater than the average indicated higher values of the respective domain [18, 45]. The validity and reliability of the instrument were previously tested and showed good agreement in most blocks, with six domains with an intraclass correlation coefficient above 0.72 (*p* < 0.05) and Cronbach’s alpha above 0.67 [30].

### Sociodemographic and anthropometric variables

Demographic characteristics included age and gender. The socioeconomic status of the participants was assessed using the Economic Classification Criteria (CCEB) of the Brazilian Association of Research Companies [49], filled in by the parents/legal guardians. The questionnaire gives different scores based on household characteristics, the level of education of the head of the family and accessibility to public services. According to the final score, participants were classified into classes: high (classes A and B1), medium (B2 and C1), and low (C2 and D-E) [49].

The anthropometric variables evaluated were weight (kg) and height (cm), measured by a digital scale (Plenna® Ice Model, São Paulo, Brazil) and portable stadiometer (Sanny® Medical, São Paulo, Brazil), according to Lohman and Roche [50]. Body mass index (BMI) was calculated using the formula (weight (kg) / height (m)^2^). From this index, adolescents’ nutritional status were classified as z-score, according to gender and age, using the BMI/age curves of the World Health Organization (WHO) [51].

### Assessment of movement behaviors

#### Physical activity, steps, sedentary behavior and sleep: Accelerometry

The ActiGraph accelerometer (GT3X Model) was used to measure light PA (LPA), moderate PA (MPA), vigorous PA (VPA), MVPA, number of steps and SB. The ActiLife software (version 6.13.4) (ActiGraph, LLC, Fort Walton Beach, USA) was used to perform all accelerometer analyses. The adolescents used the monitors on the right hip secured with an elastic belt for eight consecutive days, including during night sleep. The adolescents were instructed not to change their daily routine and the accelerometer should be removed only for aquatic activities, such as bathing and swimming. Each evaluated person received an equipment usage journal, in which they should make daily notes of the time they woke up and slept at night, in addition to the moments when the monitor was removed from the body. The first day of use (the day they received the device) was not considered in the analysis to avoid the Hawthorne Effect [52].

The accelerometer was initialized to collect data at a sampling rate of 30 Hz, with a standard filter and the data was reintegrated into 15-s *epochs*. The non-use time was defined as zero consecutive counts/minute for at least 20-min. To be included in the analysis, the participants needed to reach a minimum of 10 h.day^−1^of “time of use” [53], and at least 5 days per week, of which at least 1 day should be during the weekend. We evaluated daily charts, inclinometer data and converted this data into a Microsoft Excel comma-separated value file (.csv) to calculate the average sleep duration. To assist in the sleep time analyses, the accelerometer usage journals were also used. These sleep/awake times were used to create journals of records of the subjects and were removed from the analysis. The mean sleep duration between 8 and 10 h per day was classified as adequate sleep [54]. We adopted the cutoff points developed by Romanzini et al. [55] to classify PA and SB, validated for Brazilian adolescents, using magnitude vector and 15-s *epochs.*

Based on the weekly average, adolescents were classified into specific behavior categories. The MVPA was considered adequate when the participants performed 60 min per day [1, 2, 56]. Due to the absence of specific daily recommendations regarding the time to be allocated to each of the behaviors - LPA, MPA, VPA, SB - the 75th percentile (75thP) of the current data set was used to classify these variables, as previously used by Faria et al. [9] and Miranda et al. [57]. Thus, “adequate” times were considered when the SB was below 75^th^P and the LPA, MPA and VPA were above 75^th^P. The number of steps was classified based on the cutoff point of 11,700 steps proposed by Tudor-Locke et al. [58].

#### Screen time, cell time and sitting time: self-report

Total sitting time (TST) was defined by answering the question “*How much time do you spend sitting, talking to friends, playing cards or dominoes, talking on the phone, in traffic as a passenger, reading or studying?”*, extracted from the questionnaire “Behavior of Adolescents from Santa Catarina state”, validated for Brazilian adolescents [59]. Total TST on the 7 days of the week was estimated based on the weighted average of the sitting hours on weekdays and weekends. Due to the absence of a cutoff point for the number of sitting hours, the 75^th^P of the current dataset was used to classify this variable.

The “Portable Technologies and Mobile Internet Questionnaire” validated for Brazilian adolescents, which gauges the time spent in portable technologies, such as mobile, tablet and notebook*,* was used to evaluate the total ST and cell phone screen time (CT) [60]. The participants had to answer the question, “*On average*, how *much time do you spend accessing the internet through [technology of interest] per day?*”, for a typical weekday and a weekend day. Afterward, a score in minutes was attributed to each type of portable technology, a score for one weekday and another for a weekend day [61]. The sum of the scores in minutes for the three technologies was used to verify the total ST, and only the cellphone score in minutes were used to evaluate the CT. Total ST and CT equal to or greater than 2 h/day were considered “elevated” [62].

### Latent class manifest variables

Five latent variables extracted from the *Neighborhood Walkability for Youth* scale were selected to describe the neighborhood classes related to the different domains of the environment: land use mix-diversity, street connectivity, land use mix-access, walking/cycling facilities and pedestrian and automobile traffic safety. The variables were categorized dichotomously according to the classifications of the instrument’s environmental domains [45].

### Statistical analysis

The Statistical Package for the Social Sciences (SPSS) software for Windows, version 20.0 (IBM Corporation®, New York, USA) and the statistical software R (R Development Core Team, 2014) version 3.2.2 (“Fire Safety”) were used for the statistical analyses. The level of significance adopted was 5%.

The LCA was performed in the (poLCA) package Polytomous Variable Latent Class Analysis [63] available in the Library of Statistical Software R. The manifest variables were five factors of the NEWS-Y scale that assess the characteristics of the neighborhood. The eight factors were not analyzed simultaneously because the sample number, *n* = 309, would not avoid the scattering effect, which cannot be less than five when dividing “n” by the number of the exponential category by the number of manifest variables (W) - n / W > 5 [38]. In the present study, through tests, it was verified that 5 specific criteria interacted with each other to generate the latent variable – “Perceived Environment”. To reiterate this issue, the total sample number would not support the analysis with all 8 criteria at once. In addition, the iteration between these criterias could not be found. The LCA is an analysis based on the subject’s characteristics. Thus, tests based on hypotheses are appropriate procedures, they obtain the most adjusted, parsimonious and interpretable model.

The interpretation of the values of relative and absolute adjustments, degree of uncertainty and interpretability were used to evaluate the model’s quality. The evaluation of the most parsimonious model was based on the Akaike Information Criterion (AIC), Bayesian Information Criterion (BIC), chi-square goodness adjustment test *(Goodness of it*- χ2), entropy (evaluation of the degree of uncertainty) and maximum likelihood ratio test (G^2^). Finally, each item’s belonging probability (ρ) and the prevalence of classes (γ) allowed the analysis of homogeneity and separation of the model’s classes, and it was possible to interpretate the models from there.

After finding the latent variable, labeled “Perceived Environment”, this was considered as a result. Thus, the Kolmogorov-Smirnov test and the statistical values of skewness and kurtosis showed non normal data. Therefore, the results were presented as medians and interquartile range (IQR). The Kruskal-Wallis test was used to verify the differences in the behavior variables’ quantitative values between the three latent classes of “Perceived Environment”. The Bonferroni post-hoc test was used to verify differences between pairs of groups. This correction was calculated by dividing the total significance value (α = 0.05) adopted by the number of comparations between the three latent classes. Thus, the value of the Bonferroni correction was equal to 0.0166.

## Results

A total of 367 adolescents completed the study, but 58 were removed for inappropriate use of the accelerometer. The sample consisted of 309 adolescents, with an average age of 15.37 ± 0.57 years, of which 57% were female, 70.9% belonged to the medium SS, 78% were classified as eutrophic and 18% as overweight/obese, 52.4% met the MVPA recommendations and approximately 75% of the sample presented adequate SB. Importantly, it was considered as “adequate SB” for the different expressions of SB (ST, TST, CT, total SB) when adolescents had values below the 75^th^P cutoff point established for SB. Table 1 presents the absolute and relative frequency of the study variables.

**Table 1:** Sample characteristics according to gender

Covariates	Male (***n*** = 133)	Female (***n*** = 176)	Total (***n*** = 309)
Age group			
14	5 (3.8%)	9 (5.1%)	14 (4.5%)
15	70 (52.6%)	97 (55.1%)	167 (54.1%)
16	58 (43.6%)	70 (39.8%)	128 (41.4%)
BMI			
Low Weight	5 (3.7%)	7 (4.0%)	12 (3.9%)
Eutrophic	103 (77.5%)	138 (78.4%)	241 (78.0%)
Overweight	19 (14.3%)	26 (14.8%)	45 (14.5%)
Obesity	6 (4.5%)	5 (2.8%)	11 (3.6%)
SS			
Low	33 (24.8%)	27 (15.3%)	60 (19.4%)
Middle	62 (46.5%)	97 (55.1%)	159 (51.5%)
High	29 (21.8%)	48 (27.3%)	77 (24.9%)
-	9 (6.9%)	4 (2.3%)	13 (4.2%)
LPA			
Adequate (≥184.5 min/day)	37 (27.8%)	41 (23.3%)	78 (25.2%)
Inadequate (< 184.5 min/day)	96 (72.2%)	135 (76.7%)	231 (74.8%)
MPA			
Adequate (≥47. 85 min/day)	39 (29.3%)	39 (22.2%)	78 (25.2%)
Inadequate (< 47.85 min/day)	94 (70.7%)	137 (77.8%)	231 (74.8%)
VPA			
Adequate (≥36.5 min/day)	66 (49.6%)	12 (6.8%)	78 (25.2%)
Inadequate (< 36.5 min/day)	67 (50.4%)	164 (93.2%)	231 (74.8%)
MVPA			
Adequate (≥60 min/day)	94 (70.7%)	68 (38.6%)	162 (52.4%)
Inadequate (< 60 min/day)	39 (29.3%)	108 (61.4%)	147 (47.6%)
Steps			
Adequate (≥11,700 steps/day)	29 (21.8%)	12 (6.8%)	41 (13.3%)
Inadequate (< 11,700 steps/day)	104 (78.2%)	164 (93.2%)	268 (86.7%)
SB			
Adequate (≤740.4 min/day)	112 (84.2%)	120 (68.2%)	232 (75.1%)
Inadequate (> 740.4 min/day)	21 (15.8%)	56 (31.8%)	77 (24.9%)
Sleep Duration			
Adequate (≥8 h/day)	31 (23.3%)	48 (27.3%)	79 (25.6%)
Inadequate (< 8 h/day)	102 (76.7%)	128 (72.7%)	230 (74.4%)
ST			
Adequate (≤2 h/day)	10 (7.5%)	16 (9.1%)	26 (8.4%)
Inadequate (> 2 h/day)	123 (92.5%)	160 (90.9%)	283 (91.6%)
CT			
Adequate (≤2 h/day)	15 (11.3%)	24 (13.6%)	39 (12.6%)
Inadequate (> 2 h/day)	118 (88.7%)	152 (86.4%)	270 (87.4%)
TST			
Adequate (≤514.2 min/day)	103 (77.4%)	129 (73.3%)	232 (75.1%)
Inadequate (> 514.2 min/day)	30 (22.6%)	47 (26.7%)	77 (24.9%)

*BMI* Body mass index, *SS* Socioeconomic status, *LPA* Light physical activity, *MPA* Moderate physical activity, *VPA* Vigorous physical activity, *MVPA* Moderate to vigorous physical activity, *SB* Sedentary behavior, *ST* Screen time, *CT* Cell time, *TST* Total sitting time, - missing data.

Absolute and relative frequency of the study variables

The model tuning statistics for two to five classes are provided in Table 2. We opted for a model with three latent classes because it presented the best adjustment and interpretability values compared to the other models.

**Table 2:** Relative, absolute adjustments values and degree of uncertainty of Perceived Environment LCA models

	AIC	BIC	gl	χ^**2**^	G^**2**^	p-G^**2**^	Entropy
2 Classes	1829.8	1870.86	20	21.87	19.81	0.347	0.598
3 Classes^a^	1829.2	1892.66	14	9.27	7.38	0.812	0.772
4 Classes	1836.9	1922.78	8	5.00	4.53	0.757	0.750
5 Classes	1845.3	1953.57	2	1.38	0.95	0.499	0.588

*AIC* Akaike Information Criterion, *BIC* Bayesian Information Criterion, gl freedom degrees, χ^2^ Pearson’s chi-square test of goodness fit, G^2^ Likelihood Ratio, p-G^2^ Likelihood Ratio Test Statistics.

^a^model with best fit values. The models with six classes or more presented negative freedom degrees, so they were not shown

**Fig. 1:**
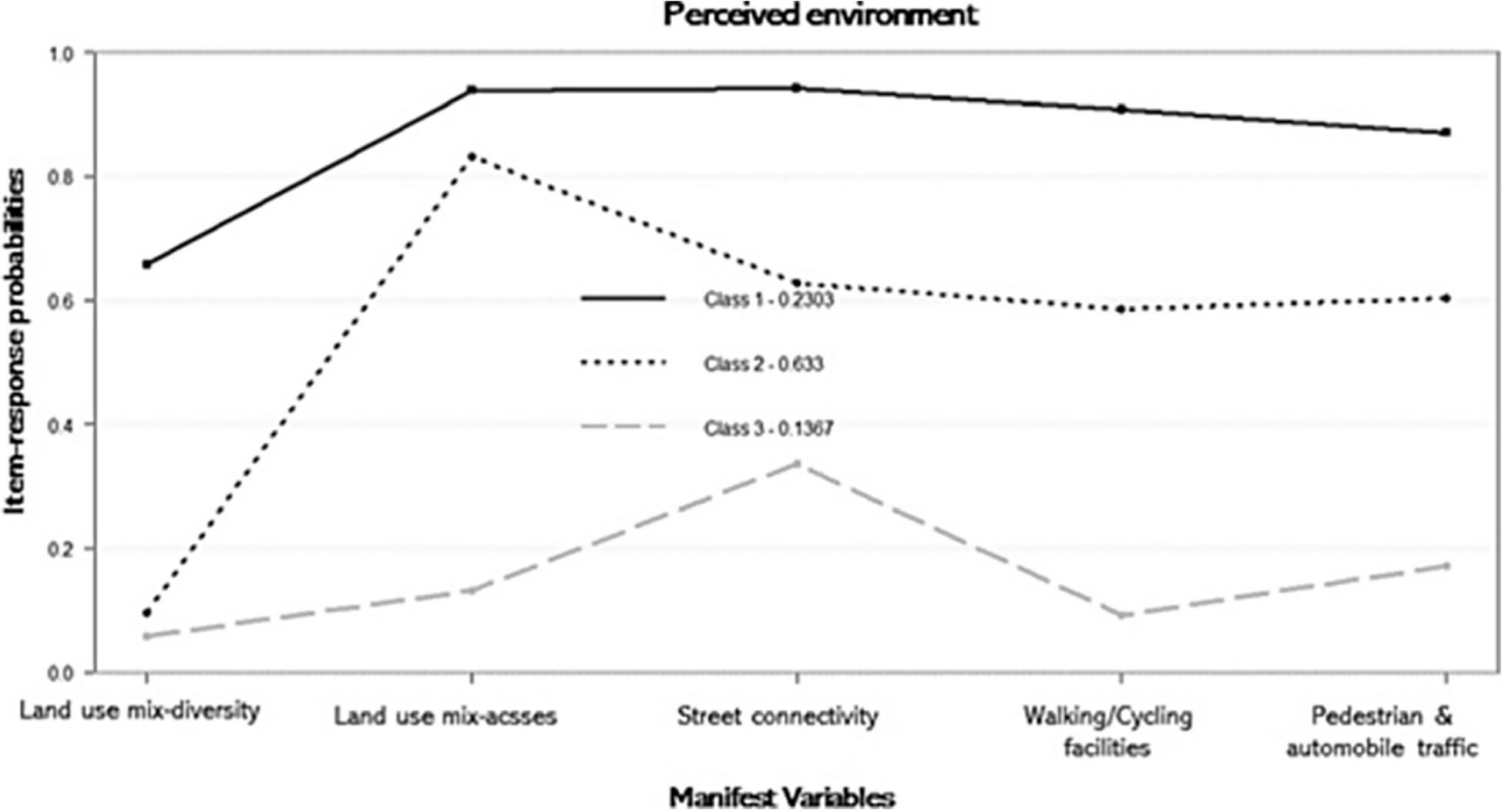
Perceived Environment LCA model. Class 1: Best Perceived Environment (γ: 23.03%), Class 2: Moderate Perceived Environment (γ: 63.33%), Class 3: Worst Perceived Environment (γ: 13.67%)

The probabilities of response to item (ρ) of the three latent classes were presented in Fig. 1. After interpreting these values, the respective latent classes were labeled as class 1, called “Best Perceived Environment”; class 2 “Moderate Perceived Environment”; and class 3, “Worst Perceived Environment”. Respectively, these latent classes presented the following prevalence values (γ) 23.03, 63.33 and 13.67%.

The PoLCA package and the adjustment criteria used evaluated the local independence criterion, which states that dependent on the latent class, the observed variables are independent. Because a complete dataset is a mixture of several latent classes, this assumption does not imply that the observed variables are independent in the sample as a whole.

The other factors of the NEWS-Y scale*, ‘*residential density’, ‘crime safety’ and ‘neighborhood aesthetics’ were tested as covariates and did not show any association with the prevalence of belonging to latent classes. The same lack of association was found when analyzing BMI and gender with the latent variable “Perceived Environment”.

On the other hand, the analysis of the association of covariates confirmed the fact that the SS is associated with the probability of belonging to the latent classes (Table 3). Compared to adolescents classified as having a high SS, those categorized with medium SS and low SS were, respectively, 3.42 (95% CI 1.62–7.21) and 4.18 (95% CI 1.66–10.50) more likely to belong to class 2, “Moderate Perceived Environment” when compared to the best class of Perceived Environment (class 1). Additionally, adolescents with low SS, compared to those with high SS, were 5.21 (95% CI 1.35–20.13) more likely to belong to the “Worst Perceived Environment” class compared to class 1, “Best Perceived Environment”.

**Table 3:** Association of covariates with the Perceived Environment LCA model

Covariates	Class 1/ Class2						Class 1/ Class3					
	β	SE	OR	CI (95%)		***P***-value	β	SE	OR	CI (95%)		***P***-value
Male^a^	1						1					
Female	0.03	0.41	1.03	0.46	2.30	0.937	0.85	0.52	2.34	0.84	6.48	0.131
High ss^a^	1						1					
Medium ss	1.23	0.38	3.42	1.62	7.21	0.006^b^	1.24	0.6	3.46	1.07	11.20	0.066
Low ss	1.43	0.47	4.18	1.66	10. 50	0.009^b^	1.65	0.69	5.21	1.35	20.13	0.039^b^
Adequate SB (75^th^P)^a^	1						1					
High SB	−0.29	0.499	0.75	0.28	1.99	0.569	−0.87	0.6	0.42	0.13	1.36	0.173
Adequate Light PA (75^th^P)^a^	1						1					
Low Light PA	−1.12	0.86	0.33	0.06	1.76	0.22	−2.37	0.9	0.09	0.02	0.55	0.021^b^
Adequate Moderate PA (75^th^P)^a^	1						1					
Low Moderate PA	0.11	0.54	1.12	0.39	3.22	0.844	−0.54	0.59	0.58	0.18	1.85	0.376
Adequate Vigorous PA (75^th^P)^a^	1											
Low Vigorous PA (75^th^P)	−0.72	0.64	0.49	0.14	1.71	0.28	0.15	0.8	1.16	0.24	5.57	0.854
Adequate MVPA^a^	1						1					
Low MVPA	−1.22	0.67	0.30	0.08	1.10	0.096	−1.09	0.7	0.34	0.09	1.33	0.148
Adequate Steps^a^	1						1					
Low Steps	−1.76	1.77	0.17	0.01	5.52	0.338	−1.54	1.78	0.21	0.01	7.02	0.404
Adequate SD^a^	1						1					
Insufficient SD	0.63	0.50	1.88	0.98	3.59	0.232	−0.37	0.54	0.69	0.24	1.99	0.504
Adequate ST^a^	1						1					
High ST	−0. 7	0.79	0.69	0.15	3.25	0.644	−1.59	0.828	0.20	0.04	1.03	0.078
Adequate TST (75^th^P)^a^	1						1					
High TST	−1.16	0.47	0.31	0.12	0.79	0.031^b^	−1	0. 6	0.37	0.11	1.19	0.121

Class 1 Best Perceived Environment, Class 2 Moderate Perceived Environment, Class 3, Worst Perceived Environment, β multinomial regression coefficient, *SE* Standard error, *OR* Odds ratio, *CI (95%)* 95% confidence interval, *75*^*th*^*P* 75th percentile, *SB* Sedentary behavior, *SS* Socioeconomic status, *PA* Physical activity, *MVPA* Moderate to vigorous physical activity, *SD* Sleep duration, *ST* Screen time, *TST* Total sitting time

^a^reference category, ^b^Significative association with LCA model latent classes

Regarding the movement behaviors evaluated by the accelerometer (LPA, MPA, VPA, MVPA, number of steps, SB, sleep duration) and self-reported method (ST, CT and TST), it was found that only the LPA level and the TST were associated with the “Perceived Environment” classes. Compared to adolescents with ‘Low LPA time’ (<75^th^P), those classified with an ‘Adequate LPA time’ (>75^th^P) were 81% less likely to belong to the “Best Perceived Environment” class than the “Worst Perceived Environment” class (OR: 0.09, 95% CI 0.02–0.55). In addition, adolescents classified with an ‘Adequate TST’ (<75^th^P), compared to those with ‘High TST’ (>75^th^P), were 69% less likely to belong to the class “Best Perceived Environment” than the class “Moderate Perceived Environment” (OR: 0.31, 95% CI 0.12–0.79).

Finally, we analyzed the difference between the quantitative BMI values and the movement behaviors variables between the three latent classes (Table 4).

**Table 4:** Variability of quantitative BMI values and movement behaviors measurements between the Environment’s classes

Quantitative variables	Class 1 (n: 52)		Class 2 (n: 218)		Class 3 (n: 39)		***p***
	Median	25thP - 75thP	Median	25^**th**^P - 75^**th**^P	Median	25^**th**^P - 75^**th**^P	
BMI (kg/m^2^)	20.20	18.65–22.27	21.00	19.07–23.20	21.10	18.90–22.80	0.532
SB (min/day)	707.50	684.60–758.70	701.25	653.30–741.25	681.40	610.40–728.60	0.059
Light PA (min/day)	147.70^c^	120.25–169.82	161.35	130.77–184.45	168.10^c^	129.90–202.50	0.022^a^
Moderate PA (min/day)	38.20	29.40–47.70	35.45	28.20–47.32	36.80	27.60–54.30	0.381
Vigorous PA (min/day)	18.90	13.02–31.72	25.00	14.77–38.50	19.20	12.30–29.40	0.160
MVPA (min/day)	61.65	46.62–77.07	62.55	44.80–81.42	54.90	42.80–90.70	0.891
Number of Steps	8353.20	6905.47–9967.07	8314.60	6541.85–10,130.27	7649.60	5797.70–10,653.30	0.689
SD (hours/day)	7.60	7.30–8.00	7.40	6.80–8.00	7.40	6.90–8.10	0.161
ST (hours/day)	5.80	3.42–10.40	7.90	4.90–10.90	5.90	3.10–9.00	0.038^a^
CT (hours/day)	4.75	2.60–9.07	6.95	4.10–10.00	5.30	2.40–9.00	0.035^a^
TST (min/day)	486.50^bc^	351.00–604.50	377.00^b^	277.75–491.00	360.00^c^	236.00–549.00	0.001^a^

Class 1 Best Perceived Environment, Class 2 Moderate Perceived Environment, Class 3 Worst Perceived Environment, *BMI* Body mass index, *PA* Physical activity, *MVPA* Moderate to vigorous physical activity, *SD* Sleep duration, *ST* Screen time, *CT* Cell time, *TST* Total sitting time, *min/day* minute per day, *hours/day* hours per day, *25*^*th*^*P* 25th percentile, *75*^*th*^*P* 75th percentile

^a^Significative *p*-value (*p* < 0.05) Kruskal-Wallis test

^b^Significative *p*-value (*p* < 0.016) of Bonferroni post-roc teste between class 1 and class 2

^c^Significative *p*-value (*p* < 0.016) of Bonferroni post-roc teste between class 1 and class 3

The results confirmed that adolescents in the class of “Worst Perceived Environment” presented higher LPA time values than the class of “Best Perceived Environment” (*p* = 0.011). In addition, the TST presented higher median values in the class “Best Perceived Environment” than in the classes “Moderate Perceived Environment” (*p* = 0.001) and “Worst Perceived Environment” (*p* = 0.006). Also, there was a variation in the values between the “Perceived Environment” classes in the ST and CT variables, however, this difference has not been confirmed by the Bonferroni post-hoc test.

## Discussion

This study aimed to identify adolescents’ latent classes based on neighborhood’s built environment characteristics through the NEWS-Y scale*,* using LCA as an approach. The findings contribute to a better understanding of the association between neighborhood classes and adolescents’ movement behaviors in a small town. It was possible to identify three latent classes in the best fit model, based on 5 NEWS-Y factors*,* which generated a latent variable representing adolescents’ “Perceived Environment”.

During the research, three studies with children and adolescents [20, 29, 33] and a study [37] with adults that used LCA to classify the built environment were found. Two of these studies acquired environmental information through georeferenced data [20, 29], one study [33] combined GIS data with information from the NEWS*-Y* scale, and Adams et al. [37] only used subjective measures as the present study.

Few studies have examined the combined effect of multiple, perceived and co-current environmental factors, and even less examined it in adolescents. A study [64] that had similar characteristics to the present study, but used latent profile analysis, identified neighborhood profiles, combining information from adult’s NEWS scale with the NEWS-Y scale, through the perception of parents regarding the environment, and related to 6- to 12-year-old children’s MVPA. Corroborating with the findings in children [64] and adults [37], the present study’s results demonstrate that using a set of environmental attributes measured only by self-report can contribute to a better understanding of neighborhood types. Additionally, it is worth mentioning that NEWS is a simple and inexpensive measurement tool that can facilitate a more in-depth assessment of neighborhood environment patterns [36].

It was possible to observe the classes separation based on the neighborhood attributes (Fig. 1). Class 1, labeled “Best Perceived Environment” was considered the best class, had a prevalence of 23.03% and presented the highest probability values for the attributes (moderate values for land use mix-diversity and very high - above 90% - for land use mix-access, street connectivity, ease for walking/cycling and for pedestrian and automobile traffic safety). Class 2, labeled “Moderate Perceived Environment”, had the highest prevalence (63.33%), with moderate values for the attributes (low land use mix-diversity, high land use mix-access and moderate for street connectivity, ease for walking/cycling and pedestrian and automobile traffic safety). Finally, class 3, labeled “Worst Perceived Environment”, had the lowest prevalence (13.67%) and presented very low probability values for all neighborhood attributes researched. The ‘land use mix-diversity’ neighborhood attribute presented the lowest value in the three classes, especially in classes 2 and 3, demonstrating that the commercial establishments and facilities are distant from the residences in the neighborhoods where the participants live.

Kurka et al. [64] evaluated children’s latent profiles from two metropolitan regions of the United States. Despite the proportion differences between the cities, some similarities in the prevalence and characteristics were observed (e. g., ease of walking and safety) in relation to the present study. A higher prevalence of adolescents in the “Moderate Perceived Environment” class was observed in the same way as occurred in the previously mentioned study, which also presented a high number of children in profile 2 “Moderate Walking”, with a prevalence of 43 and 35.2%. Also, a smaller number of participants living in the “Worst Perceived Environment” class was observed, similar to the lower prevalence (23.6%) in profile 1 “Low for Walking”, from the same study.

Further, it was possible to test environmental covariates’ influence – ‘crime safety’, ‘neighborhood aesthetics’ and ‘residential density’ between the “Perceived Environment” classes. However, no association was found between the classes, possibly because the variables tested were homogeneous among the neighborhoods investigated, which was also observed when these variables were tested in the model. Because this is a small town, the neighborhoods have minor differences in residential and aesthetic density; most neighborhoods have houses or buildings with few floors. They do not have natural attractions and green spaces that may differ between neighborhoods, aside from being a city with a low crime rate is small compared to large urban centers.

Non-association was also demonstrated when the covariates gender and BMI were tested. Differences in the perception of neighborhood characteristics between boys and girls were not observed, explaining the non-association of classes with the gender variable. Regarding BMI, this can be explained because the medians between classes were very close (Table 3), demonstrating that the type of neighborhood had no interference in the sample’s body indexes. Additionally, it was noted that most of the participants were eutrophic (Table 1).

The results showed an association between the classes of “Perceived Environment” with the SS. When comparing adolescents classified with high SS, those categorized with medium and low SS were more likely to belong to class 2 than class 1. Adolescents with low SS compared to those with high SS were more likely to belong to class 3 than class 1, demonstrating that participants with high SS are in the best class. The findings support the well-established knowledge that neighborhoods with a high SS have a better structure regarding commercial facilities, access to commerce and security [65]. Wall et al. [33] verified similar patterns when comparing the SS among six neighborhood classes. The two classes identified with high SS had a higher number of parks and recreation areas; the class with average SS contained an urban residential with parks. Still, with a low safety perception and little traffic, the three most socioeconomically disadvantaged classes present a lower safety perception.

The influences of the “Perceived Environment” classes were tested in 10 variables related to movement behaviors (LPA, MPA, VPA, MVPA, number of steps, SB, sleep duration, ST, CT and TST), and we decided to discuss only the variables that presented association. There was association between the neighborhood classes only for AFL and TST (Table 3). The non-influence of neighborhood classes in all PA levels and SB expressions was not exclusively observed in the present study. Mcdonald et al. [20] did not find any evidence of a neighborhood class effect on total PA, SB, and ST of adolescents. For the authors, these results suggest that the association between the built environment and PA in general and adolescent PA, more specifically, is difficult to interpret, given the diversity of challenges in this research area. Because there is a lack of a clear definition of neighborhoods and consistent measures of the built environment, it may be necessary to expand the “area” considered as the individual’s neighborhood. For example, when considering adolescents, it should include also the area surrounding the school and other places outside the home where they spend a significant part of their time (friends’ houses, squares, sports clubs).

It was possible to observe that the magnitude and, sometimes, the direction of the associations between the characteristics of the environment built with PA and with the SB vary between studies. This variation may be due to differences in sampling, measurement of different combinations of environmental factors (e. g., community design, recreational environments, social environment, school environment) and how the results were operationalized (e. g., total PA, MVPA episodes, active transportation).

Further analyzing it is observed that the LPA differed among the classes of “Perceived Environment”. Adolescents classified with an ‘Adequate LPA Time’ had a lower chance of belonging to the “Best Perceived Environment” class than the “Worst Perceived Environment” class (Table 3). This fact was confirmed by the significant difference in LPA time in minutes between classes (Table 4), where those who lived in perceived environments with “Worst Perceived Environment” engaged about 20 min a day more in LPA compared to those living in places of “Best Perceived Environment”. Although some studies show that favorable physical environments, such as higher residential density, urban sidewalks and connected streets, close to parks and commerce [29–31, 66] are associated with longer PA time and lower SB, this was not observed in the best neighborhood class for LPA in the present study. Land use mix-diversity and land use mix-access notably showed very low proportions in class 3. Both, respectively, refer to the time of commuting home to the facilities and shops of the neighborhood and the ease of access to these locations. Hence, the longer time in LPA can be explained by the fact that the adolescent has to move for a longer time to his neighborhood facilities.

Added to this, the SS may have a more significant influence on LPA time since adolescents with low SS had a greater chance of belonging to the worst class of environment. Furthermore, the LPA is more related to everyday activities. This socioeconomic reality can cause adolescents in this class to become more involved in household chores, such as doing dishes, caring for their younger siblings, and some need to work outside the home to help with family income. In addition, previous studies indicate that domestic activities are responsible for one-third of the children’s non-school activities [67] and one-fifth of the adolescents’ non-school activities [68]. It is also important to highlight the longer time in AFL of participants from the worst class, since recent studies have demonstrated LPA health benefits [10, 69].

The current analysis also showed an association between the “Perceived Environment” classes and the TST (Table 3). Adolescents classified with an ‘Adequate TST’ were less likely to belong to the “Best Perceived Environment” class than the “Moderate Perceived Environment” class. Also, a significant difference was observed for TST in minutes between these classes (Table 4). Adolescents in the “Moderate Perceived Environment” class were seated 109.5 min per day less than those of “Best Perceived Environment” class.

Although class 2 presents high values for land use mix-access and moderate values for street connectivity, ease for walking/cycling and for pedestrian and automobile traffic safety, it has very low values for land use mix-diversity. Perhaps the adolescents’ residences are further from the facilities and shops, which obliges them to walk more. Another possible explanation is the autonomy and independence that is acquired during adolescence, where more freedom to choose activities and become more independent and mobile is granted, with fewer parental restrictions [70], these factors that were not controlled in the present study may have favored adolescents of the moderate class to spend less TST.

It was also observed that the adolescents who belong to the “Worst Perceived Environment” class, were seated for roughly 126.5 min a day less than the residents of the best neighborhood class. It is noticed that living in a place with a larger structure for walking, street connectivity and safety did not make the adolescents of the best neighborhood class involved in more significant LPA time and lower TST. These are intriguing findings that differ from the literature, where it is generally expected that people living in better-structured environments become more involved in PA and spend less time sitting [28, 30, 36]. The fact that adolescents in the “Best Perceived Environment” class are also the ones with the best SS, possibly means that they use a car as a means of transportation, have greater supervision and monitoring of their studying time, and have opportunities to engage in extracurricular activities, such as language courses, which could justify the greater TST and shorter time in LPA.

Studies that sought to investigate the influence of SB’s neighborhood classes, especially in TST, are limited, which hinders the findings’ interpretation and comparability. Different results were found in adolescents living in walkable neighborhoods, who reported less television time and less time in vehicles [28]; and adolescents who lived in neighborhoods with diversified land use had less time watching television [71], however, both studies examined the influence of individual environmental factors in SB and were performed in very different realities from those of the present study.

This study’s strengths include the application of LCA to create adolescents’ classes of neighborhood perceived environment, where it was possible to identify a model with three latent classes. It is worth highlighting that this is the first Brazilian latent class study that uses neighborhood environment characteristics to evaluate adolescents’ behaviors. Even when reviewing international literature, few publications jointly analyze the influence of environmental characteristics in the different levels of PA and different expressions of adolescents SB. Another important consideration is conducting this study in a small, countryside town since studies with these characteristics usually occur in large urban centers. Thus, the present study’s information may encourage other researchers to investigate the influence of the small-town neighborhood environment’s characteristics on adolescents’ movement behaviors.

On the other hand, some limitations were observed. Firstly, the exclusive use of a subjective measure to evaluate the characteristics of the neighborhood environment. However, studies previously conducted with children and adults [33, 37], using LCA, confirm a strong association of neighborhood classes based on self-reported environmental variables with PA. In addition, we think it is important to know how people are perceiving the environment in which they are inserted. Second, the dichotomization of the built environment characteristics to facilitate the understanding of the information may have led to some information losses. Third, it is worth mentioning that the results could have been different if a cutoff point different from the 75^th^P for the variables LPA, MPA, VPA, SB and TST was selected. However, there is no consensus on a cutoff point for these variables. However, it should be noted that the 75^th^P was applied in other LCA studies [9, 57] and can be a useful method for comparing teens with their peers. Fourth, multilinearity tests were not performed for the covariates because these were associated with the model separately, which means that the evaluation of the maximum likelihood statistic (p-G2) values was carried in a particular way for each covariate tested. Therefore, the lack of covariates multicollinearity tests can be considered a limitation of the model. Finally, the difficulty of comparing our results to those of other studies. This fact can be attributed to the lack of a consensus in the literature on which neighborhood variables should be used and the different types of measures used to evaluate the environment. In addition, the results of the present study based on neighborhood classes are difficult to compare directly with previous studies that examined the individual characteristics of the environment or with studies that only evaluated total PA and MVPA and did not evaluate time in the various expressions of SB.

Based on the presentation and discussion of the results, the need to carry out further studies that use methods of classification of the neighborhood, such as LCA is apparent, because it explicitly addresses the complex web of characteristics to which individuals are exposed in a neighborhood, which can affect their PA and SB. Further research using LCA in other small towns will allow verification of the existence of patterns. In addition, “neighborhood class creation” using statistical model approaches, even based on self-report measures, can reveal neighborhood characteristics and their real needs, generating information so that public policies can implement approaches in communities, such as interventions in built environments, creating or improving environments conducive to PA, and propose strategies that can be effective in promoting active lifestyles and overcoming environmental barriers related to PA.

## Conclusion

The present study found a model with three latent classes derived from the neighborhood environment of adolescents’ attributes. It was possible to observe an association between the classes and the SS. Adolescents with medium SS and low SS were more likely to belong to the “Moderate Perceived Environment” class than to the “Best Perceived Environment” class. While those with low SS were more likely to belong to the “Worst Perceived Environment” class than the “Best Perceived Environment” class. As for movement behaviors, it was observed that adolescents in the “Worst Perceived Environment” class accumulated a longer time in LPA compared to the best neighborhood class. In addition, adolescents residing in the “Moderate Perceived Environment” class were involved in a lower TST than those residing in the “Best Perceived Environment” class.

Based on the above, applying the LCA strategy to other samples with these environmental measures is important to determine whether neighborhood class and similar relationships with movement behaviors are reproducible. Also, knowledge of neighborhood class models in a city may be more effective in positively influencing adolescent movement behaviors than information on the built environment’s individual attributes.

## Supplementary Information

**Additional file 1.**

## Data Availability

The data set generated during the present study is available online at https://data.mendeley.com/datasets/zj5nz4rt9x/draft?a=01fbfb0d-4b11-4ff9-955d-43ae937ebf61.

## Abbreviations

BMI: Body mass index
SS: Socioeconomic status
PA: Physical activity
SB: Sedentary behavior
LPA: Light physical activity
MPA: Moderate physical activity
VPA: Vigorous physical activity
MVPA: Moderate to vigorous physical activity
ST: Screen time
CT: Cell time
TST: Total sitting time
LCA: Latent class analysis
GIS: Georeferenced data
NEWS-Y: Neighborhood Walkability Scale for Youth
WHO: World Health Organization
