# Supplementary material for: Adolescent’s movement behaviors and built environment: a latent class analysis
Source: BMC Public Health. 2021 Oct 25;21:1937. doi: 10.1186/s12889-021-11974-4 (PMC8547093; doi:10.1186/s12889-021-11974-4)
Supplement: Supplementary file 1 — Additional file 1. [file 12889_2021_11974_MOESM1_ESM.zip › Complementary_Material_NEWS_CCE_ESM.docx]

**Neighborhood Environment Walkability Scale for Youth (NEWS-Y)**

The scale presents 73 questions divided into eight domains/subscales, which are answered according to adolescents’ neighborhood perception. Except for the first subscale, the other’s questions are related to the adolescents’ residence surroundings characteristics, considering the distance covered by a 10 to 15 minutes walk. Next, each subscale is described in detail, with an example of the questions, score, metrics, and classification, according to Rosenberg et al. (2009).

**Subscale 1: Land use mix-diversity and Recreation facilities (question 1 to 34)**

The first subscale questions assess the young person’s perception of the travel time from their residence to establishments and places of recreation in the neighborhood. Examples of the questions are: How long would it take you to walk to shops, offices, and other destinations (e.g., market, post office, school)? About how long would it take you to walk to types of recreation destinations (e.g., swimming pool, basketball court, parks)? Responses are provided on a 6-point Likert scale: 1 = 1 to 5 min; 2 = 6 - 10 min, 3 = 11 - 20 min, 4 = 20 - 30 min, 5 = 31+ min and 6 = do not know/do not have.

Initially, each adolescent’s answers were summed to evaluate this subscale. The classification was obtained by comparing the sum of each adolescent’s responses with the sum of the mean scale, which is equivalent to 119 points. Scores below average indicate greater environment use, i.e., greater proximity between the house and the various locations. Conversely, values above 119 indicate less environment and greater distance from the house to the various locations.

**Subscale 2: Residential density (question 35 to 42)**

The second subscale’s eight questions refer to the adolescents’ perception of the different housing types, their amount, and neighborhood spatial distribution. For example: In your neighborhood, how many residences are separate or stand-alone one-family homes? Responses are provided on a 5-point Likert scale: 1 = none, 2 = few, 3 = some, 4 = many and 5 = all.

Initially, the means of the answers to the eight questions for each adolescent were calculated to evaluate this subscale. The classification was determined by comparing the mean of the answers and the scale’s mean value, equivalent to 3. Thus, values above 3 indicate high residential density, while values below 3 indicate low residential density.

**Subscale 3: Land use mix-access (question 43 to 48)**

The third subscale’s six statements aim to verify the adolescent’s perception of the ease of access to some existing structures, for example: Stores are within easy walking distance of my home; From my home, it is easy to walk to a transit stop; the streets in my neighborhood are hilly. Again, the answers are provided on a 4 points Likert scale: 1 = strongly disagree, 2 = somewhat disagree, 3 = somewhat agree, and 4 = strongly agree.

To evaluate this subscale, the means of the six statements’ answers for each adolescent were initially calculated. Then, the classification was determined by comparing the answers’ mean and the scale’s mean value in this domain, which is equivalent to 2.5. Thus, values above 2.5 indicate better access to some neighborhood structures, while values below 2.5 indicate worse access.

**Subscale 4: Street connectivity (question 49 to 51)**

The three statements of the fourth subscale assess the adolescent’s perception of the neighborhood’s streets and the ease of going from one point to another. These are examples of the statements: the streets in my neighborhood do not have many cul-de-sacs (dead-end streets); There are many different routes for getting from place to place in my neighborhood. Again, the answers are provided on a 4 points Likert scale: 1 = strongly disagree, 2 = somewhat disagree, 3 = somewhat agree, and 4 = strongly agree.

Initially, to evaluate this subscale, the means of the answers to the three statements should be calculated for each adolescent. Then, the classification was determined by comparing the answers’ mean and the scale’s mean value in this domain, which is equivalent to 2.5. Thus, values above 2.5 indicate better street connectivity, while values below 2.5 indicate worse street connectivity.

**Subscale 5: Walking/cycling facilities (question 52 to 54)**

The three statements of the fifth subscale assess the adolescent’s perception of places to walk / bike in the neighborhood. Examples of the statements: There are sidewalks on most streets in my neighborhood; there is grass/dirt between the roads and the sidewalks. The answers are provided on a 4 points Likert scale: 1 = strongly disagree, 2 = somewhat disagree, 3 = somewhat agree, and 4 = strongly agree.

To evaluate this subscale, initially, the means of the three statements’ answers for each adolescent should be calculated. Then, the classification was determined by comparing the answers’ mean and the scale’s mean value in this domain, equivalent to 2.5. Thus, values above 2.5 indicate better infrastructure for pedestrians/ cyclists, while values below2.5 indicate worse infrastructure for pedestrians/cyclists.

**Subscale 6: Neighborhood Aesthetics (question 55 to 58)**

The four statements of the sixth subscale assess the young person’s perception of the neighborhood’s aesthetics. Examples of the statements: There are trees along the streets in my neighborhood; There are many interesting things to look at while walking in my neighborhood. The answers are provided on a Likert scale of 4 points: 1 = strongly disagree, 2 = somewhat disagree, 3 = somewhat agree, and 4 = strongly agree.

To evaluate this subscale, initially, the means of the four statements’ answers for each adolescent should be calculated. The classification was determined by comparing the answers’ mean and the scale’s mean value in this domain, equivalent to 2.5. Thus, values above 2.5 indicate better aesthetics, while values below 2.5 indicate worse aesthetics.

**Subscale 7: Pedestrian and car traffic safety (question 59 to 66)**

The eight statements of the seventh subscale assess the young person’s perception of traffic safety. Examples of the statements: There is so much traffic along nearby streets that it makes it difficult or unpleasant to walk; traffic speed is usually slow; the roads have good lighting. The answers are provided on a Likert scale of 4 points: 1 = strongly disagree, 2 = somewhat disagree, 3 = somewhat agree, and 4 = strongly agree.

To evaluate this subscale, initially, the means of the answers to the 8 questions for each adolescent should be calculated. The classification was determined by comparing the answers’ mean and the scale’s mean value in this domain, equivalent to 2.5. Thus, values above 2.5 indicate better-perceived safety, while values below 2.5 indicate worse perceived security.

**Subscale 8: Crime security (question 67 to 73)**

The seven statements of the eighth subscale assess the adolescent’s perception of crime in the neighborhood. Examples of the statements are: There is a high crime rate in my neighborhood; I am worried about being outside alone around my home (like in the yard, driveway, or apartment common area) because I am afraid of being taken or hurt by a stranger. The answers are provided on a Likert scale of 4 points: 1 = strongly disagree, 2 = somewhat disagree, 3 = somewhat agree, and 4 = strongly agree.

To evaluate this subscale, initially, the means of the seven statements’ answers for each adolescent should be calculated. The classification was determined by comparing the answers’ mean and the scale’s mean value in this domain, equivalent to 2.5. Thus, values above 2.5 indicate higher crime perceptions /lower security, while values below 2.5 indicate lower crime perceptions /greater security.

**Referência:** Rosenberg D, Ding D, Sallis JF, Kerr J, Norman GJ, Durant N, et al. Neighborhood Environment Walkability Scale for Youth (NEWS-Y): Reliability and relationship with physical activity. Prev Med. 2009;49(2–3):213–218. http://dx.doi.org/10.1016/j.ypmed.2009.07.011.

**ECONOMIC CLASSIFICATION CRITERION**

The Economic Classification Criterion (ECC) allowed identifying Brazilian families’ real consumption potential, through variable indicators of permanent income, using the Household Budget Survey of the Brazilian Institute of Geography and Statistics (IBGE) (ABEP, 2016).

The criterion’s questions refer to: 1) working electronic items in the house, 2) water system used in the residence, 3) if the part of the street is asphalted/paved or land/gravel, and 4) head of the family’s instruction degree.

By fulfilling the criterion, the person reports each item’s amount in the household and answers the other questions. In the criterion, there is a points system that is assigned to each question. For the family’s economic classification, it is necessary to replace the values of each item and answered questions; then these are summed. From this score, the family will be classified into one of the six socioeconomic strata named A, B1, B2, C1, C2, and D-E. In the present study, three categories were used: high (classes A and B1), medium (B2 and C1), and low (C2 and D-E).

**Referência:** Associação Brasileira de Empresas de Pesquisas - (ABEP). (2016). Available from: http://www.abep.org/criterio-brasil. Accessed on Jan 20, 2019.
